# Alloyed Airways: Chromium, Beryllium, and Titanium in the Lungs of an Aerospace Veteran

**DOI:** 10.7759/cureus.58392

**Published:** 2024-04-16

**Authors:** Mark A Potesta, Aaron Brown, Akram Shibani

**Affiliations:** 1 Medicine, Lake Erie College of Osteopathic Medicine, Bradenton, USA; 2 Pulmonary and Critical Care, Ascension St. Vincent's Southside Hospital, Jacksonville, USA

**Keywords:** obstructive lung disease, restrictive lung disease, heavy metal pneumoconiosis, titanium, beryllium exposure, chromium, pneumoconiosis

## Abstract

Pneumoconiosis is a form of interstitial lung disease (ILD) that commonly occurs secondary to occupational or environmental exposures. This is an emerging disease as there are many potential forms of pathologic insults. Further adding to the complication is that clinical symptomatology secondary to pneumoconiosis can have long latent periods, as repetitive exposure over years leads to long-standing inflammation and subsequent irreversible damage. Exposure to asbestos, coal, silica, aluminum, talc, hay, and many more agents has the potential to cause pneumoconiosis. This case highlights a veteran, who made his career working with heavy metals such as chromium, beryllium, and titanium in the aerospace defense industry. This case discusses high-risk occupations, a workup for suspected pneumoconiosis, management, and the mechanism of lung injury underlying the three aforementioned pathologic agents. In each case of pneumoconiosis, a thorough history is essential, and diagnoses are made via the incorporation of the patient’s historical risk factors, pulmonary function test (PFT) findings, and high-resolution chest computed tomography (HRCT).

## Introduction

Pneumoconiosis is a form of pulmonary disease associated with the inhalation of particulate matter, most commonly from occupational exposure. Inhalation of these particles leads to the development of pulmonary inflammation. Chronic levels of lung inflammation can lead to pulmonary fibrosis and eventually death from respiratory and cardiac failure as a sequela of pulmonary fibrosis [1]. Pneumoconiosis has multiple subcategories based on pathological insult. Examples of pathological insult include, but are not limited to, silica, coal, talc, asbestos, benign or inert forms from iron, tin, barium, chronic beryllium disease (CBD), and giant cell interstitial pneumonia (GIP) [2]. About 527,500 people annually are living with pneumoconiosis [1]. In 2017, the incidence was reported to be over 60,000 [1]. Since 2015, approximately 21,000 new deaths each year due to pneumoconiosis have been reported [1]. There are two proposed mechanisms of disease. The first proposed mechanism of the disease involves antigen-specific immune responses, while the second mechanism of the disease involves nonspecific innate immune responses that lead to reactive oxygen species-induced inflammation [3].

Beryllium, titanium, and chromium are three such heavy metals that are known causative agents of pneumoconiosis that are further explored in this case report. Beryllium is a heavy metal with many favorable characteristics that have led to its incorporation within the technology sector. Beryllium is commonly machined and processed into alloy and ceramic products, which are used in the electronics, aerospace, machining, and nuclear weapons manufacturing industries. This increased use of beryllium has led to an increased prevalence of CBD, a form of pneumoconiosis. Beryllium, when inhaled, becomes an antigen and is subsequently presented by antigen-presenting CD4+ T cells to initiate an immune inflammatory response [3].

Titanium particles known to cause lung disease are commonly found in two forms: metallic titanium or titanium dioxide (TiO_2_). Metallic titanium is commonly used in the aerospace industry, whereas TiO_2_ is commonly used in products such as paints, pigments, and cosmetics. The majority of titanium exposure is to TiO_2_, particularly a subset of TiO_2_ known as titanium nanoparticles (TNP) [4]. TNP is less than 20 nanometers in size, making it small enough to enter the pulmonary interstitium and create an inflammatory response. This inflammatory response is mediated by reactive oxygen species [5]. Chromium is another heavy metal known to cause pathologic insult. This is particularly troublesome when it is accumulated in large quantities in the lungs, liver, and kidneys of humans. High-level chromium exposure is most commonly seen in three major workspaces: welders working in stainless steel-producing industries, workers in the electroplating industry, and workers involved in chromate production. Inhalation is the major route by which workers in these industries are exposed to chromium. The damage to lung tissue from chromium exposure is linked to a large amount of reactive oxygen species-mediated oxidative stress [6]. Common symptoms of pneumoconiosis include persistent fatigue, worsening dyspnea, and a nonproductive cough [1].

The first line evaluation for heavy metal pneumoconiosis in those exposed is chest radiography. However, those who are symptomatic and whose chest radiography is without evidence of the disease should receive high-resolution computed tomography (HRCT) to better visualize parenchymal involvement. Highly suspicious signs of pneumoconiosis on chest radiography include small rounded radiopaque nodules distributed throughout the lung parenchyma. Ultimately, diagnosis of heavy metal pneumoconiosis is highly dependent on both a positive clinical history, indicating exposure, and a suspicious radiographic presentation [2]. Currently, the only treatment capable of extending life in patients with pneumoconiosis is lung transplantation [1].

## Case presentation

A 69-year-old male, with a 30-pack-year history, presented to the pulmonary office with chronic dyspnea on exertion and new onset right-sided pleuritic chest pain that started two months ago. His occupational history is significant for working as a career aircraft mechanic in the armed forces. The patient reported no prior history of asthma, emphysema, or other chronic diseases.

**Figure 1:**
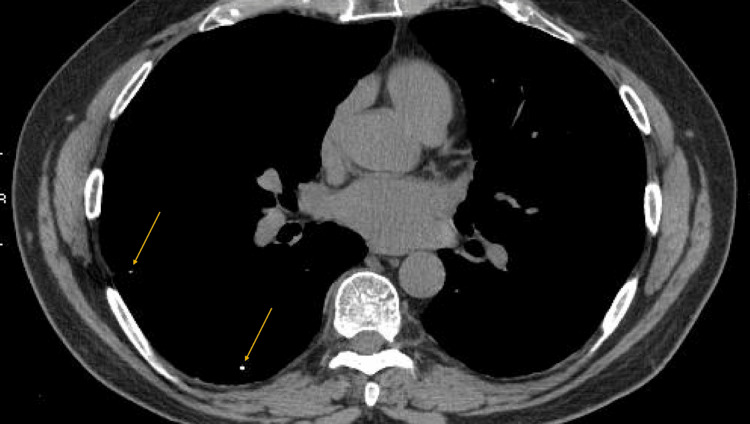
High-resolution CT, exhibiting small radiopaque nodules scattered throughout the patient’s lung.

The initial workup included pulmonary function testing (PFT) and high-resolution CT (HRCT). His PFTs were consistent with clinically significant reactive airway disease, as seen by a 9% increase in his forced expiratory volume in one second (FEV1) post bronchodilator. This finding was most pronounced in the mid-flow region. Although the patient did not have a previously established diagnosis of emphysema or reactive airway disease, his PFTs suggested a mixed picture of the aforementioned diseases and interstitial lung disease (ILD). With multiple disease processes contributing to the patient's PFTs, the patient's entire clinical history, as well as the overall disease picture, had to be accounted for. His HRCT was suggestive of subtle, lower lobe predominant sub-pleural reticular nodular findings with scattered areas of tiny radiopaque nodules (Figure 1). These findings could be correlated with a component of his PFTs, which showed mild diffusion impairment, with his corrected diffusion capacity of the lungs for carbon monoxide (DLCO) being 74% of predicted, likely suggestive of ILD. His clinical symptomatology was out of proportion to the size of the radiopaque nodules seen on CT. As the patient’s PFTs also showed that he was bronchodilator responsive, he was started on Breo Ellipta (fluticasone/vilanterol) one inhalation daily, as well as albuterol as needed, both of which did not alleviate his symptoms. Further workup included an echocardiogram, right heart catheterization, and autoimmune serology. His echocardiogram showed a normal left ventricular ejection fraction of 50-55% and a right ventricular systolic pressure of around 40 mmHg (20-30 mmHg), suggestive of moderate pulmonary hypertension. Subsequent right heart catheterization showed pulmonary artery pressures of 35/7/17 (20-30/8-15/10-20), pulmonary capillary wedge pressure (PCWP) of 5 mmHg (4-12 mmHg), and left ventricular end-diastolic pressure (LVEDP) of 8 mmHg (4-12 mmHg), and his pulmonary vascular resistance (PVR) was 2 woods unit (< 2 woods unit). Additionally, his autoimmune serology was unrevealing.

The patient’s unresolved symptomatology coupled with his mixed picture PFTs (Table 1), nonspecific HRCT findings, symptomatology out of proportion to findings, and poor response to medical therapy prompted obtaining a detailed occupational history. At this time, the patient revealed a career history as a mechanic for aircraft in the armed forces. He noted he was frequently exposed to heavy metals, in particular, beryllium, titanium, and chromium. Bronchoscopy samples in this case were unwarranted due to the particular small nature of the metals. However, his occupational history and HRCT findings strongly suggested that heavy metals were a significant contributing source to his lung disease. This case outlines the importance of obtaining a detailed occupational history when working on heavy metal pneumoconiosis as a potential cause of lung disease.

**Table 1:** Patient's pulmonary function testing. FEV1: Forced Expiratory Volume in One Second; FVC: Forced Vital Capacity; TLC: Total Lung Capacity; RV: Residual Volume; DLCO: Diffusing Capacity of the Lungs for Carbon Monoxide Note: Normal spirometry findings have an FEV1/FVC ratio > 0.70 and both FEV1 and FVC greater than 80% of predicted. Bronchodilator responsiveness is indicative of reactive lung disease, such as asthma. Reference ranges for pulmonary function testing (PFT) results are influenced by age, sex, and height. The entire clinical picture should be accounted for when interpreting the diagnosis.

Parameter	Measurement	Predicted %
FEV1	3.6%	97
FVC	5.1%	102
FEV1/FVC ratio	74%	-
Bronchodilator response	+ 9%	-
TLC	98%	-
RV	98%	-
RV/TLC ratio	40%	-
DLCO	71%	-
Corrected DLCO	74%	-

## Discussion

To work a patient up for a heavy metal pneumoconiosis, a thorough history, coupled with correlation with PFT and HRCT findings, is essential. As heavy metal pneumoconiosis is a form of ILD, workup follows a typical sequence [7]. Initially, PFTs are obtained, followed by serology for connective tissue disease and HRCT. Connective tissue disease can manifest as a form of ILD, so ruling this out through serology (antinuclear antibodies and rheumatoid factor) helps narrow the differential [7].

The patient’s HRCT was evaluated for findings of usual interstitial pneumonia (UIP), in which classic features consist of reticular opacities in basal and peripheral distribution, traction bronchiectasis, and honeycombing in the sub-pleural region [7]. If the patient’s lung disease is not explained in the setting of UIP, further evaluation of HRCT findings for sarcoidosis, berylliosis, hypersensitivity pneumonitis, carcinomatosis, and other forms of occupational lung disease/heavy metal pneumoconiosis should be accounted for [7]. Only if the patient’s lung disease cannot be explained by the former should they undergo diagnostic bronchoscopy with broncho-alveolar lavage and trans-bronchial lung biopsy [7].

ILD findings on pulmonary function testing should be consistent with a restrictive pattern [8]. Furthermore, a restrictive lung disease pattern that is indicative of ILD should have a forced expiratory volume in one second to forced vital capacity (FEV1/FVC) ratio of ~0.70 (normal) and a low diffusion capacity of the lungs for carbon monoxide (DLCO) (less than 75% of predicted) [8]. Conversely, in obstructive lung diseases such as asthma and emphysema, the FEV1/FVC ratio is typically < 0.70 (low), with asthma having a bronchodilator response and not emphysema. If the patient is not broncho-dilatory responsive in the setting of an obstructive lung disease further evaluation of DLCO is necessary. If the DLCO is low (less than 75% of predicted), this is indicative of emphysema. If it is high or normal, this is indicative of chronic bronchitis or asthma [8].

Although the patient had not been diagnosed with emphysema or asthma in the past, his PFTs were suggestive of a mixed picture of lung disease. This was likely due to a combination of social factors (30-pack-year history), a component of mild asthma (seen by broncho-dilatory response), as well as occupational/environmental influence (seen by the 74% FEV1/FVC ratio, as well as the corrected DLCO being 74% of the predicted). The patient’s HRCT findings were suggestive of subtle, lower lobe predominant sub-pleural reticular nodular findings and scattered areas of tiny radiopaque nodules. Although these findings had sub-pleural predominance that could be suggestive of UIP, there was also the component of the tiny radiopaque nodules, scattered throughout. As the patient’s lung overall picture of lung disease, as well as the nodules, could not be explained by UIP or rheumatologic disease, reevaluation of the patient's occupational history was warranted. It was at this time that the patient revealed a career history as a mechanic for aircraft in the armed forces. He noted that he was frequently exposed to heavy metals, in particular, beryllium, titanium, and chromium. Due to the small and diffuse nature of the radiopaque nodules and the patient’s occupational history providing a clear explanation for the findings, further diagnostic bronchoscopy was not warranted. The management of occupational lung disease is largely symptomatic; however, biologics are used for severe disease, and steroids are used during flares [1]. Occupational lung disease is an emerging diagnosis. Significant research is not yet available; however, anti-fibrotic agents such as pirfenidone have shown promise [1].

Occupational lung disease includes pneumoconiosis (ILDs), hypersensitivity pneumonitis, bronchiolitis, byssinosis, and occupational asthma [2]. The pathophysiology underlying pneumoconiosis results from inhalation of particles and repetitive exposure. Patients with lung disease secondary to heavy metal particulates, typically do not present in a clear-cut fashion. To complicate things further, patients may present with a mixed clinical picture when factoring in cigarette use. Initially, patients with pure heavy metal pneumoconiosis may present with nonspecific features such as dry cough and shortness of breath.

The latency period between initial beryllium exposure and disease manifestation typically varies from three months to 30 years. Involvement of the bronchial tree can lead to a clinical picture similar to asthma. Beryllium is lighter than aluminum and six times stronger than steel [9]. It is also commonly alloyed with copper, aluminum, and nickel [9]. Patients at highest risk for developing this form of lung disease are those who have had occupational exposure via machinery, electronic industry, construction and shipyard, and aerospace/defense industry (such as this patient). The disease develops as beryllium elicits a cell-mediated hypersensitivity reaction [9]. Subsequent repetitive exposure to the antigen leads to the upregulation of pro-inflammatory mediators and the development of non-caseating granulomas, which are seen in histology [9]. Diagnosis can be made via history of beryllium exposure, imaging consistent with chronic beryllium disease, compatible histology, and positive beryllium lymphoproliferative test [9].

Titanium has two primary classifications, which either fall into the category of metallic titanium or TiO_2_. The metallic form of titanium is commonly used in the aerospace industry, as well as other industrial industries. On the other hand, TiO_2_ is typically associated with commercially available products such as paints, pigments, and cosmetics [4]. TiO_2_ can further be classified into titanium nanoparticles (TNP), which are poorly soluble and small enough to access the pulmonary interstitium [5]. More than 90% of the world’s titanium ore is turned into TiO_2_ or TNP, while 5% is used for metallic titanium [4]. The size of the nanoparticles, typically < 20 nanometers, plays an important role in the reactivity, affecting cellular uptake and lung injury. The hallmark of injury is via reactive oxygen species (ROS). The inhaled nanoparticles lead to an inflammatory response, and overwhelming antioxidant defenses, leading to a proliferative and genotoxic response [5].

Chromium exposure at high levels most commonly occurs through occupational exposure [6]. Careers involving welding, stainless steel, electroplating, and aerospace [6,10]. One of the most common routes of chromium exposure is through inhalation. Chromium-associated disease causes ROS production and subsequent toxicity in major organs such as the lungs, liver, and kidneys and can also cause widespread dermatitis [6,10]. Additionally, it has been linked to lung cancer, as well as cardiomyopathies [6,10]. Although ROS production is the primary mechanism of injury, chromium exposure has also been seen to cause alterations in DNA methylation, thus causing injury at a genetic level [6].

## Conclusions

Pneumoconiosis is a form of ILD secondary to occupational or environmental exposure. When working up pneumoconiosis as part of a differential diagnosis for ILD, the physician needs to take a thorough occupational history to account for risk factors. It is also essential to incorporate HRCT and PFT findings, as well as rule out other potential causes of ILDs, such as connective tissue disease, before ruling in pneumoconiosis. As an emerging field in which disease manifestation has the potential for decades of latency, further research is needed to determine the spectrum of potential inciting agents, as well as their long-lasting effects. There is research, however, supporting the three agents in this case, beryllium, titanium, and chromium, as having the potential to cause ILD. While treatment options are not plentiful and complete remission is not likely outside of lung transplant, advances in anti-fibrotics have shown promise.
